# Kynurenine, a Tryptophan Metabolite That Increases with Age, Induces Muscle Atrophy and Lipid Peroxidation

**DOI:** 10.1155/2019/9894238

**Published:** 2019-10-13

**Authors:** Helen Kaiser, Kanglun Yu, Chirayu Pandya, Bharati Mendhe, Carlos M. Isales, Meghan E. McGee-Lawrence, Maribeth Johnson, Sadanand Fulzele, Mark W. Hamrick

**Affiliations:** Medical College of Georgia, Augusta University, Augusta, GA 30912, USA

## Abstract

The cellular and molecular mechanisms underlying loss of muscle mass with age (sarcopenia) are not well-understood; however, heterochronic parabiosis experiments show that circulating factors are likely to play a role. Kynurenine (KYN) is a circulating tryptophan metabolite that is known to increase with age and is a ligand of the aryl hydrocarbon receptor (Ahr). Here, we tested the hypothesis that KYN activation of Ahr plays a role in muscle loss with aging. Results indicate that KYN treatment of mouse and human myoblasts increased levels of reactive oxygen species (ROS) 2-fold and KYN treatment *in vivo* reduced muscle size and strength and increased muscle lipid peroxidation in young mice. PCR array data indicate that muscle fiber size reduction with KYN treatment reduces protein synthesis markers whereas ubiquitin ligase gene expression is not significantly increased. KYN is generated by the enzyme indoleamine 2,3-dioxygenase (IDO), and aged mice treated with the IDO inhibitor 1-methyl-D-tryptophan showed an increase in muscle fiber size and muscle strength. Small-molecule inhibition of Ahr *in vitro*, and Ahr knockout *in vivo*, did not prevent KYN-induced increases in ROS, suggesting that KYN can directly increase ROS independent of Ahr activation. Protein analysis identified very long-chain acyl-CoA dehydrogenase as a factor activated by KYN that may increase ROS and lipid peroxidation. Our data suggest that IDO inhibition may represent a novel therapeutic approach for the prevention of sarcopenia and possibly other age-associated conditions associated with KYN accumulation such as bone loss and neurodegeneration.

## 1. Introduction

The population of people 60 years of age or older is expected to increase from 8% of the world's population in 2013 to an estimated 21% of the world's population by 2050. This increase means that in the next 40 years, there will be more than 2 billion people over 60 years of age [1]. Extending the health span of the world population so that aged people can remain disease-free and independent is an important step toward easing the burden of medical costs and increasing the quality of life. Several factors contribute to age-related decline in independence with sarcopenia, the loss of muscle mass and power, being one of the most important. Sarcopenia occurs in over one-third of people over 70 years of age [2]. Sarcopenia is a multifactorial disease with unknown causes. Sarcopenia can be characterized by several broad symptoms: generalized muscle atrophy, increases in systemic cellular reactive oxygen species (ROS), mitochondrial dysfunction, replacement of muscle fibers with fibrotic factors and fat, and degradation of neuromuscular junctions [3]. Currently, there are no FDA-approved medications for sarcopenia [4].

Heterochronic parabiosis experiments have shown that circulating factors from young blood can help regenerate aged and injured muscle [5]. This suggests that some circulating factors in old blood may be harmful, or conversely that factors in young blood may be helpful, to aging muscle. Kynurenine (KYN) is a circulating tryptophan metabolite that increases with age and is implicated in several age-related disorders including neurodegeneration, osteoporosis, and inflammation [6, 7]. KYN is metabolized from tryptophan by two major enzymes: tryptophan 2,3-dioxygenase (TDO) in the liver and indoleamine 2,3-dioxygenase (IDO) extrahepatically [8]. IDO is induced by several inflammatory cytokines including IL-6, IL-1*β*, and interferon-gamma (IFN*γ*) [9]. An increase in IDO activity has been linked to an increased mortality rate in humans [10], and frailty is associated with a marked increase in the KYN/TRP ratio [11].

We hypothesized that an increase in KYN with age contributes to muscle atrophy and oxidative stress. We also tested the hypothesis that inhibition of IDO to decrease the production of KYN in aged mice might attenuate muscle atrophy and oxidative stress. We used 1-methyl-D-tryptophan (1-MT), a specific antagonist of IDO that has been shown to deplete murine KYN levels [12]. We further sought to understand the mechanism behind KYN-induced ROS. Several previous studies have shown that KYN is a ligand for the aryl hydrocarbon receptor (Ahr), a xenobiotic drug response transcription factor [13, 14] that is associated with age-related changes in vascular tissues [15] and skin [16]. We also tested the hypothesis that KYN activation of Ahr with aging could contribute to sarcopenia by increasing oxidative stress and reducing muscle mass and strength. We used a known inhibitor of KYN-induced Ahr activation, CH-223191, to test the effect of KYN's activation of Ahr in skeletal muscle [17, 18].

## 2. Materials and Methods

### 2.1. Animal Experimental Design

All aspects of the animal research were conducted in accordance with the guidelines set by the Augusta University Institutional Animal Care and Use Committee (AU-IACUC) under an AU-IACUC-approved animal use protocol. For KYN and 1-MT treatment studies, 6-8-month-old (young adult) and 22-24-month-old (aged) female C57BL/6 mice were obtained from the aged rodent colony at the National Institute of Aging (NIA, Bethesda, MD, USA). Female mice were chosen due to higher rates of sarcopenia observed in women [19]. For Ahr studies, young adult male and female Ahr-knockout mice were obtained from Taconic (#9166). For KYN treatment and Ahr-KO studies, mice received daily intraperitoneal (I.P.) injections of vehicle (VEH; phosphate-buffered saline, PBS) or L-kynurenine (Sigma; #K8625) at 10 mg/kg body weight for 4 weeks (*n* = 10 per group). For 1-MT studies, 22-24-month-old (aged) C57BL/6 mice were used. Mice were split into 3 groups (*n* = 20 per group): aged VEH (sterile H_2_O, 0.20 mL injection), aged low 1-MT (10 mg/kg 1-MT, 0.20 mL injection), and aged high 1-MT (100 mg/kg 1-MT, 0.20 injection). No acute adverse effects were detected with injected KYN or 1-MT. KYN doses were chosen based on previous work in bone [20]. 1-MT doses were chosen based on toxicology work on 1-MT in rats and dogs [21]. Mice were euthanized using CO_2_ overdose followed by thoracotomy according to AU-IACUC-approved animal protocols. The right quadriceps was fixed in 10% formalin and stored in 70% ethanol for paraffin embedding and histology. The left quadriceps was frozen in liquid nitrogen for protein and gene expression analysis, and the right tibialis anterior was used for an Amplex™ Red assay immediately.

### 2.2. Cell Culture

C2C12 cells were obtained from ATCC (ATCC® CRL-1772™), and primary human myoblasts were obtained from Gibco. Both cell lines were cultured in Dulbecco's modified Eagle's medium (DMEM) (Gibco, USA) containing 10% fetal bovine serum (Gibco, USA) and 1% penicillin-streptomycin (Gibco, USA). Cells were seeded in the media and maintained at 37°C in a 5% CO_2_ cell incubator (Thermo, USA) until 70%-80% confluence. KYN concentrations of 1 *μ*M and 10 *μ*M were chosen based on serum concentrations found in healthy vs. pathological humans [10].

### 2.3. Histological Staining

Quadriceps femoris muscles were fixed in 10% buffered formalin, paraffin embedded, and sectioned at 6-8 *μ*m. Sections were deparaffinized and rehydrated, and nonspecific binding was blocked by 0.3% H_2_O_2_ in TBS. Sections were then incubated overnight at room temperature with rabbit polyclonal anti-laminin (dilution 1 : 1000, Sigma-Aldrich, USA) and rabbit anti-4HNE (Alpha Diagnostic HNE11-S, dilution 1 : 50) and ChromPure Bovine IgG antibodies (Jackson, 001-000-003, dilution 1 : 50). The laminin sections were washed with phosphate-buffered saline (PBS, pH 7.4) and incubated for 1 h at room temperature with a goat anti-rabbit Alexa Fluor 488-conjugated secondary antibody (Invitrogen, A11008). 4HNE and IgG sections were incubated with a polyvalent secondary antibody, followed by *streptavidin* solution (Abcam ab93697). 4HNE and IgG were visualized using DAB Liquid Chromogen Solution (Sigma D3939) and counterstained with hematoxylin (Fisher 245-677). Muscle fiber size was determined by creating a grid on ImageJ and measuring one muscle fiber in each voxel. One section was selected at random from each mouse (*N* = 10 per group in WT mice, *N* = 9 per group in Ahr-KO mice), and 10 muscle fibers per section were measured; then, an average fiber diameter per mouse was calculated. Percentage of 4HNE-positive staining was measured using Photoshop. All measurements were performed by an investigator blinded to group assignment.

### 2.4. Proteomics and Western Blot

In order to select protein candidates, proteomics were run on three quadriceps muscle samples from the following groups: young KYN, young VEH, old 1-MT (high dose), and old VEH. Quadriceps muscles were homogenized and protein was run at the Augusta University proteomics core using an Orbitrap Fusion™ Tribrid™ mass spectrometer. All proteins with a two-fold or greater difference were chosen from each group, and the protein candidate that was differentially expressed in the separate treatment groups was identified. For western blots, human myoblasts were lysed in radioimmunoprecipitation assay (RIPA) buffer (Tecnova) containing 1% protease inhibitor cocktail (Sigma). Protein concentrations were obtained using a BCA Protein Assay Kit (Sigma). Protein was run in SDS-polyacrylamide gels and transferred using electrophoresis onto a nitrocellulose membrane (Bio-Rad). Blots were incubated with a rabbit polyclonal anti-mitochondrial very-long chain acyl-CoA dehydrogenase (VLCADm) antibody (ab155138) overnight at 4°C. After washing with 1× PBS and blocking with 5% milk in 1× PBS, blots were incubated with an HRP-conjugated anti-rabbit secondary antibody (Santa Cruz Biotechnology) for 1 hr, followed by developing with the ECL Plus Western Blotting Detection System (GE Healthcare). Chemiluminescence signals were captured on autoradiographic blue films (BioExpress). Films were scanned, and the densitometric values for the proteins of interest were corrected using *β*-actin with ImageJ Software (NIH).

### 2.5. MTT Assay

Myoblast viability after KYN treatment was determined using the MTS assay (Promega CellTiter 96® AQueous One MTS Cell Proliferation Assay). C2C12 myoblasts were plated in a 96-well plate at an initial seeding density of 2500 cells/cm^2^ or 5000 cells/cm^2^. After 24 hours, myoblasts (*N* = 8 per group) were treated with 1× PBS, 5 *μ*M KYN, 10 *μ*M KYN, or 40 *μ*M KYN for 24 hours and 48 hours. After treatment, myoblasts were washed with PBS 2× and 20 *μ*L of MTS assay buffer (MTS, CellTiter 96® AQueous One Solution Reagent, Promega) was added in 100 *μ*L of media. The myoblasts were kept at 37°C in a humidified 5% CO2 incubator for 2 hours; then, optical density was read at 490 nm.

### 2.6. Amplex Red Assay

A fluorometric method was used to measure H_2_O_2_ in myoblasts treated with KYN using an Amplex Red assay kit. C2C12 myoblasts were plated in a 96-well plate at an initial seeding density of 5000 cells/cm^2^. After 24 hours, myoblasts (*N* = 6 per group) were treated with 1× PBS, 1 *μ*M KYN, or 10 *μ*M KYN for 24 hours. After treatment, media were removed and cells were suspended in sodium phosphate buffer (0.05 M, pH 7.4, 100 mL) and plated in triplicate in a flat-bottom 96-well plate. The reaction was started by adding an Amplex™ Red reagent, horseradish peroxidase, and p-tyramine. After 30 min incubation in the dark, the production of H_2_O_2_ was quantified at 37°C in a multidetection microplate fluorescence reader (Synergy H1, BioTek Instruments) based on the fluorescence generated at an emission wavelength of 590 nm upon excitation at 545 nm. The specific final fluorescence emission was calculated against a standard curve of H_2_O_2_ incubated simultaneously.

### 2.7. PCR Array Plates

Quadriceps muscles from three young VEH mice and three young KYN-treated mice were sonicated, and RNA was isolated using the TRIzol reagent (Invitrogen) according to the manufacturer's instructions. Total RNA was purified using an RNeasy Mini Kit (Qiagen). 1 *μ*g of total RNA was then reverse transcribed using the First-Strand Synthesis Kit (Qiagen) and subsequently loaded into Skeletal Muscle Myogenesis and Myopathy RT^2^ Profiler PCR Arrays (Qiagen). PCR was run at the following conditions: 10 min at 95°C, 45 cycles of 15 s at 95°C, and 1 min at 60°C. Fold change was calculated by determining the ratio of mRNA levels to control values using the ΔCt method (2^−ΔΔCt^). All data were normalized to an average of six housekeeping genes: Actb, B2m, Gapdh, Gusb, Hsp90ab1, and MGDC. PCR conditions used are as follows: hold for 10 min at 95°C, followed by 45 cycles of 15 s at 95°C and 60 s at 60°C.

### 2.8. Muscle Function Testing

Muscle peak twitch was measured using a whole mouse testing apparatus (1300A, Aurora Scientific Inc., Aurora, ON, Canada) and a force transducer (Aurora Scientific Inc., Canada). This apparatus provides torque measurements in milliNm of tetanic contraction while the animal is alive and with normal vasculature, innervation, and muscle orientation. Animals were maintained under anesthesia through a CO_2_ and oxygen breathing cone. Animals were placed on a 37°C platform, and the right hind foot was stabilized to a foot lever with cloth tape at 20° of plantar flexion. Needle electrodes were placed under the skin below the knee to stimulate the peroneal nerve. Muscle peak twitch was then recorded. Tetanic contractions (350 ms train) at 10 to 250 Hz were elicited to obtain a force-frequency curve, with a 2-minute rest between each contraction. Results of single stimulations were collected in torque (milliNm). From torque measurements, the specific muscle force is obtained by normalizing the absolute force values (milliNm) to the animal's body weight. Peak muscle twitch values were selected from each animal.

### 2.9. Statistical Analysis

For all experiments with more than 2 groups, an ANOVA and a post hoc LSD test for differences between means were used. Results from the Ahr-KO studies were compared to WT experiments using a two-factor ANOVA (SPSS). Results for all experiments with 2 groups were determined using a 2-sample independent *t*-test to compare differences between means of groups. A minimum significance level of 5% (*P* < 0.05) was used. The datasets generated during and/or analyzed during the current study are available from the corresponding author on reasonable request.

## 3. Results

### 3.1. KYN Treatment Increases ROS Levels in Mouse and Human Myoblasts

**Figure 1:**
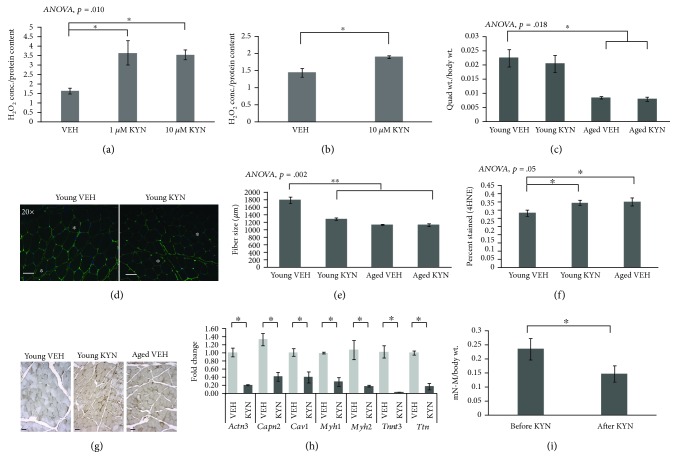
Increased measures of oxidative stress, reduction in muscle fiber size, and reduced muscle strength after KYN treatment *in vitro* and *in vivo*. (a) H_2_O_2_ levels were significantly increased in mouse C2C12 myoblasts after 24 hours of 1 *μ*M and 10 *μ*M KYN treatment compared to VEH treatment. *N* = 6/group. (b) H_2_O_2_ levels in human primary myoblasts were increased significantly after 24 hours of 10 *μ*M KYN treatment compared to VEH treatment. *N* = 5/group. (c) After 4 weeks of treatment with KYN or VEH, young female mice did not have a reduction in quadriceps mass compared to body mass. (d, e) Quadriceps fiber size was significantly reduced with KYN treatment in young female mice, visualized with laminin staining (scale bar 100 *μ*m). (f, g) Young female mice treated with KYN had a significant increase in lipid peroxidation, measured by 4HNE staining, when compared to young VEH mice or old VEH mice. (h) Changes in the expression of muscle structural protein genes in quadriceps muscles from young female mice treated with KYN compared to young VEH. (i) Young female mice lost significant peak muscle strength after 4 weeks of KYN treatment. *N* = 10/group for C-I. Data are presented as mean ± s.e.m. ^∗^*P* < 0.05, ^∗∗^*P* < 0.01, and ^∗∗∗^*P* < 0.001. Representative muscle fibers are marked with stars in d and g.

In order to determine the effect of KYN on ROS production in muscle cells, we measured H_2_O_2_ levels from C2C12 myoblasts and primary human myoblasts that were treated with KYN for 24 hours. H_2_O_2_ levels were measured using an Amplex™ Red assay. In C2C12 myoblasts, H_2_O_2_ was increased two-fold with KYN treatment at only 1 *μ*M (Figure 1(a)). The higher dose (10 *μ*M) produced no further increase beyond the lower 1 *μ*M dose. H_2_O_2_ was significantly increased in primary human myoblasts treated with 10 *μ*M KYN (Figure 1(b)). KYN did not alter C2C12 myoblast viability at 5 *μ*M, 10 *μ*M, or 40 *μ*M after 24 or 48 hours of treatment (Supplemental [Supplementary-material supplementary-material-1]).

### 3.2. KYN Treatment Decreases Muscle Fiber Size, Expression of Muscle Structural Muscle Protein Genes, and Peak Strength in Young Female Mice

Young (6-8 mo.) and aged (22-24 mo.) female C57/BL6 mice were given intraperitoneal injections with KYN (10 *μ*g/kg Sigma k3750) or VEH (1× PBS) daily for 4 weeks to test the effect of increased KYN on skeletal muscle *in vivo*. Quadriceps weight relative to body weight was not significantly reduced in KYN-treated muscle compared to vehicle controls (Figure 1(c)), and muscle fiber size was significantly lower in young mice treated with KYN compared to VEH (Figures 1(d) and 1(e)). Positive 4HNE staining, indicative of lipid peroxidation from oxidative stress, was significantly increased in young mice treated with KYN compared to young and old controls (Figures 1(f) and 1(g)). PCR arrays for muscle atrophy genes did not show significant changes in ubiquitin ligase gene expression with KYN, but the expression of myosin heavy chain genes was significantly decreased with KYN treatment (Figure 1(h)). Functional, *in vivo* assessment of muscle contractile force showed that young KYN-treated mice lost significant peak muscle strength after 4 weeks of treatment (Figure 1(i)).

### 3.3. 1-MT Treatment Resulted in Attenuated Muscle Atrophy and Enhanced Muscle Strength in Old Female Mice

**Figure 2:**
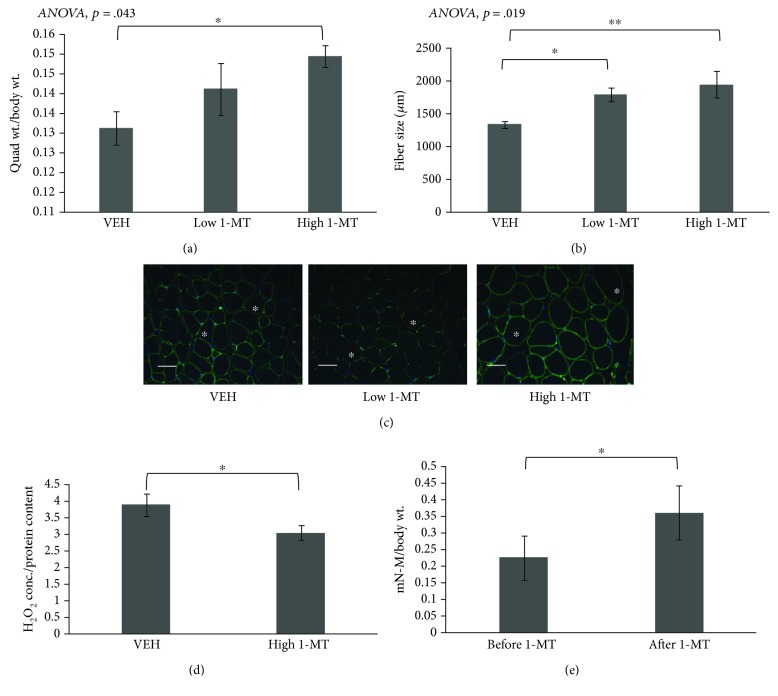
Oxidative stress and muscle morphology changes after 1-MT treatment in aged female mice. (a) Quadriceps muscle mass was significantly increased in mice treated with high-dose 1-MT compared to VEH. (b, c) Muscle fiber size was significantly increased in mice treated with low- and high-dose 1-MT compared to VEH, visualized with laminin staining. Representative muscle fibers are marked with stars. Scale bar: 100 *μ*m. (d) H_2_O_2_ levels were significantly decreased in mouse quadriceps muscles with 1-MT treatment. (e) Aged mice gained significant peak muscle strength after 4 weeks of 1-MT treatment. *N* = 20/group. Data are presented as mean ± s.e.m. ^∗^*P* < 0.05, ^∗∗^*P* < 0.01, and ^∗∗∗^*P* < 0.001.

To test the effect of IDO inhibition on skeletal muscle *in vivo*, aged female C57/BL6 mice were given intraperitoneal injections of 1-MT at a low dose (10 mg/kg) and high dose (100 mg/kg) or VEH (1× PBS) daily for 4 weeks. Quadriceps weight relative to body weight was significantly increased in mice treated with a high dose of 1-MT compared to vehicle-treated mice (Figure 2(a)). Muscle fiber size in both treatment groups was significantly increased compared to that in vehicle-treated mice (Figures 2(b) and 2(c)). H_2_O_2_ levels were significantly lower in aged mice treated with a high dose of 1-MT compared to VEH (Figure 2(d)). Muscle peak contractile force was significantly higher in mice treated with the high dose of 1-MT compared to VEH controls (Figure 2(e)).

### 3.4. Proteomic Analysis of KYN-Treated and 1-MT-Treated Skeletal Muscle Reveals Differential Expression of Very-Long Chain Acyl-CoA Dehydrogenase

**Figure 3:**
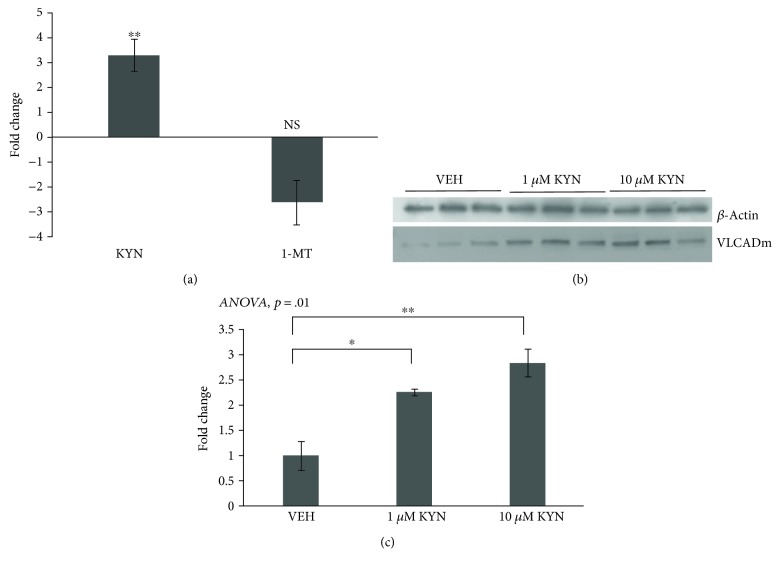
Mitochondrial very long-chain acyl-CoA dehydrogenase protein is differentially expressed with KYN or 1-MT treatment of female mice *in vivo* and human myoblasts *in vitro.* (a) Proteomics run on quadriceps muscle homogenates from young mice treated with KYN or aged mice treated with 1-MT showed that VLCADm was significantly increased with KYN treatment in young mice. (b) Representative western blots from primary human cells that showed a significant increase in VLCADm with KYN treatment. (c) VLCADm western blot results were normalized to *β*-actin controls and quantified. Data are presented as mean ± s.e.m. ^∗^*P* < 0.05, ^∗∗^*P* < 0.01, and ^∗∗∗^*P* < 0.001.

Quadriceps tissue from female young mice treated with KYN and aged mice treated with 1-MT were used for proteomic analysis. Both groups were compared to age-matched VEH-treated controls. Proteins that were decreased with KYN and increased with 1-MT or increased with KYN and decreased with 1-MT were selected. The top protein candidate that was differentially expressed with KYN and 1-MT compared to age-matched VEH controls was VLCADm (Figure 3(a)). VLCADm was significantly increased at 1 *μ*M and 10 *μ*M KYN treatment of primary human myoblasts compared to VEH controls (Figures 3(b) and 3(c)).

### 3.5. AHR Inhibition *In Vitro* and AHR Deficiency *In Vivo* Do Not Inhibit KYN-Induced ROS Accumulation

**Figure 4:**
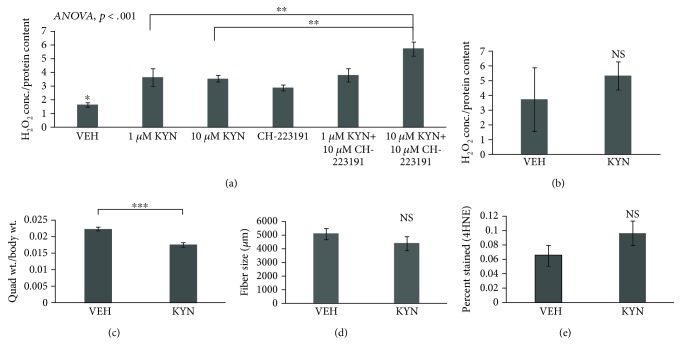
Effects of KYN with inhibition of Ahr *in vitro* and global knockout of Ahr *in vivo*. (a) Treatment of myoblasts with KYN and the small-molecule Ahr inhibitor CH-223191 simultaneously increased H_2_O_2_ significantly compared to KYN alone (*N* = 6/group). (b) H_2_O_2_ from quadriceps of Ahr-knockout mice treated with KYN was increased slightly but not significantly compared to that from VEH. (c) KYN treatment of Ahr-knockout mice significantly decreased quadriceps mass. (d) Fiber size of quadriceps muscles from Ahr-knockout mice decreased slightly but not significantly with KYN treatment. (e) 4HNE staining of quadriceps muscles increased slightly but not significantly with KYN treatment. Data are presented as mean ± s.e.m. ^∗^*P* < 0.05, ^∗∗^*P* < 0.01, and ^∗∗∗^*P* < 0.001. (b–d) Pooled male and female: *N* = (5 M + 4F per group).

To test if a KYN-induced increase in ROS is mediated through the activation of Ahr, C2C12 mouse myoblasts were treated with CH-223191, a specific small-molecule antagonist of Ahr in the presence and absence of 1 *μ*M and 10 *μ*M of KYN. All KYN-treated groups had significantly higher H_2_O_2_ than the VEH group, regardless of whether the Ahr inhibitor was present. The groups treated with 10 *μ*m KYN and CH-223191 had significantly higher H_2_O_2_ levels than the group treated with KYN alone (Figure 4(a)). To further explore the relationship between Ahr and KYN in skeletal muscle, male and female global Ahr-KO mice (6-8 months old) were treated with KYN (10 mg/kg) or VEH (1× PBS) by intraperitoneal injections daily for 4 weeks. There was a not significant difference in H_2_O_2_ in Ahr-KO mice compared to VEH. Quadriceps muscle weight was significantly lower in Ahr-KO mice with KYN treatment compared to VEH treatment (Figure 4(c)). Muscle fiber size was not significantly different in Ahr-KO mice with KYN treatment compared to VEH. 4HNE staining was not significantly different in Ahr-KO mice with KYN treatment compared to VEH. No sex differences were observed; data shown in Figure 4 are pooled. A two-factor ANOVA was run with genotype (WT, Ahr KO) and treatment (VEH, KYN) as the two factors to determine whether the absence of the Ahr receptor would significantly impact lipid peroxidation assessed by 4HNE staining. We found no significant genotype∗treatment interaction for either muscle fiber size (*F* = 0.10, *P* = 0.75) or 4HNE staining intensity (*F* = 0.54, *P* = 0.47), indicating that the loss of the Ahr receptor did not significantly alter the response of muscle to KYN treatment.

## 4. Discussion

The cellular and molecular processes leading to sarcopenia are incompletely understood. A number of factors may contribute to loss of muscle mass and strength with age including lack of physical activity, dietary protein deficiency, circulating inflammatory cytokines, and oxidative stress. Skeletal muscle has the ability to activate antioxidant proteins to quickly repair exercise-induced oxidative damage; however, these mechanisms are attenuated with age, causing an imbalance in ROS [22]. Accumulation of ROS has in particular been suggested to induce age-related declines in muscle [23, 24]. We addressed this knowledge gap by examining the cellular and molecular mechanisms underlying the age-associated accumulation of reactive oxygen species. KYN is a circulating tryptophan metabolite that increases with age and is correlated with frailty [11] and increased mortality in older adults [10]. Elevated circulating levels of KYN are also found to be associated with osteoporosis and Alzheimer's disease [7, 25, 26]. In the presence of IFN*γ*, IDO converts tryptophan to KYN [8] and IFN*γ* and IDO activity have both been shown to increase with age [9, 18].

As IDO activity is increased, the essential amino acid tryptophan is depleted from the tissue microenvironment and its metabolism is directed away from serotonin synthesis and toward the KYN pathway [8, 9]. Our study addressed the hypothesis that KYN contributes to the progression of sarcopenia. We found that KYN did not decrease quadriceps weight in young mice, but did decrease muscle fiber size. This discrepancy is most likely due to an increase in noncontractile tissue (fat or fibrotic tissue) within the muscles of KYN-treated mice (source). PCR array data identified several structural muscle proteins that were downregulated with KYN treatment. The PCR data suggest that reduction in fiber size with KYN treatment may be due to a loss of protein anabolism rather than an increase in protein catabolism. These results are consistent with those of several groups that have shown that protein anabolism is impaired with aging [27–30]. We observed an increase in the oxidative stress markers H_2_O_2_ and 4HNE in young mice treated with KYN. We noted that the levels of these oxidative stress markers were similar to those of aged mice and did not continue to increase with further KYN treatment in old mice. We speculate that the effects of KYN reach a threshold, such that additional exogenous KYN in animals that already have high KYN levels may yield no effects (kynurenine resistance), but further work to test this hypothesis is needed. We also demonstrated that inhibition of IDO helped to preserve muscle mass and function in aged mice, further suggesting that the kynurenine pathway plays an important role in muscle health.

KYN is a ligand of the aryl hydrocarbon receptor (Ahr) and is involved in immunosuppression [13]. Previous work on skin and vascular aging have suggested a potential for Ahr activation in the aging of various tissue types [15, 16]. Ahr is a xenobiotic drug response element that acts as a transcription factor and once it is activated stimulates the expression of Cyp1A1, which can further increase oxidative stress [13, 14]. We found that Ahr inhibition did not protect muscle cells from the detrimental effects of KYN treatment. Surprisingly, when myoblasts were treated with KYN and the Ahr small-molecule inhibitor CH-223191, there were significantly higher levels of H_2_O_2_ than with KYN alone. CH-223191 is a highly specific inhibitor capable of blocking KYN's interactions with Ahr in cells such as bone marrow-derived murine dendritic cells (BMDCs) [18]; however, until now, the effects of KYN's interaction with Ahr has not been explored in skeletal muscle. Ahr-knockout mice treated with KYN had significantly lower quadriceps weight compared to VEH-treated controls, but there were no significant differences between VEH- and KYN-treated mice in muscle fiber size, H_2_O_2_, or 4HNE staining in Ahr-knockout mice. This could again be due to a threshold effect, or possibly a protective effect of Ahr in skeletal muscle. Overall, these results suggest that KYN-induced skeletal muscle loss with age may occur through a different pathway than the one mediated by Ahr.

**Figure 5:**
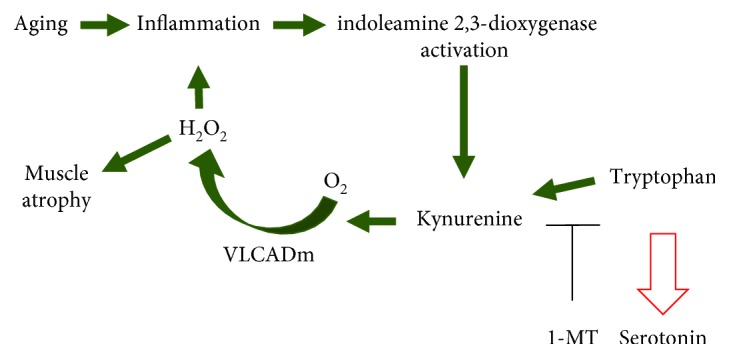
Proposed model for KYN's effects on lipid peroxidation, ROS generation, and age-related muscle atrophy. Aging causes an increase in systemic oxidative stress, and skeletal muscle antioxidant pathways are unable to meet the increased demand. Inflammatory cytokines like IFN*γ* activate IDO to convert tryptophan to KYN. KYN then induces the upregulation of mitochondrial VLCADm which degrades lipid and produces H_2_O_2_. The overexpression of ROS leads to further inflammation and muscle atrophy. 1-MT is an antagonist of IDO that can inhibit tryptophan's conversion into KYN, thereby attenuating KYN-induced tissue dysfunction with aging.

Maintenance of skeletal muscle throughout life is dependent upon a balance of protein synthesis and protein catabolism [27–30]. We examined proteomics from young mice treated with KYN, and old mice treated with 1-MT, and found that lipid peroxidation products were differentially expressed with manipulation of the KYN pathway. We identified an increase in a mitochondrial lipid peroxidation enzyme, VLCADm, as a potential downstream target mechanism for KYN's contribution to sarcopenia. VLCADm has been shown to produce H_2_O_2_ [31] (Figure 5). Furthermore, Montes et al. demonstrated that lipid peroxidation markers can serve as indicators of sarcopenia [32], and Bellanti et al. found that lipid peroxidation products form aldehyde-protein hybrids that are increased in sarcopenic adults [33]. Exercise has been previously reported to increase skeletal muscle ROS as well as VLCADm [34], suggesting that in the setting of acute inflammation transient, elevated ROS and VLCADm levels may have beneficial effects on skeletal muscle; however, it is likely that chronically elevated ROS and VLCADm resulting from prolonged KYN exposure may ultimately have detrimental effects on muscle, which would explain the previous associations among aging, inflammation, circulating KYN, VLCADm, ROS, and sarcopenia noted above (Figure 5).

## 5. Conclusion

Our work provides evidence that an increase in KYN with age may contribute to sarcopenia by causing an increase in oxidative stress. Our working model is that the lipid peroxidation resulting from chronic KYN exposure contributes to sarcopenia (Figure 5). These results provide new insights into the mechanisms underlying sarcopenia and shed new light on potential therapeutic targets for the aging population.

## Data Availability

All data used to support the findings of this study are available from the corresponding author upon request.

## Supplementary Materials

Supplementary Materials Supplemental Figure 1: the effect of KYN on C2C12 myoblast viability. (A–D) There was no change in C2C12 myoblast viability with a seeding density of 2500 cells/cm^2^ or 5000 cells/cm^2^ and 24 or 48 hours of treatment.
