# Supplementary figures and images for: Kynurenine, a Tryptophan Metabolite That Increases with Age, Induces Muscle Atrophy and Lipid Peroxidation
Source: Oxid Med Cell Longev. 2019 Oct 13;2019:9894238. doi: 10.1155/2019/9894238 (PMC6815546; doi:10.1155/2019/9894238)

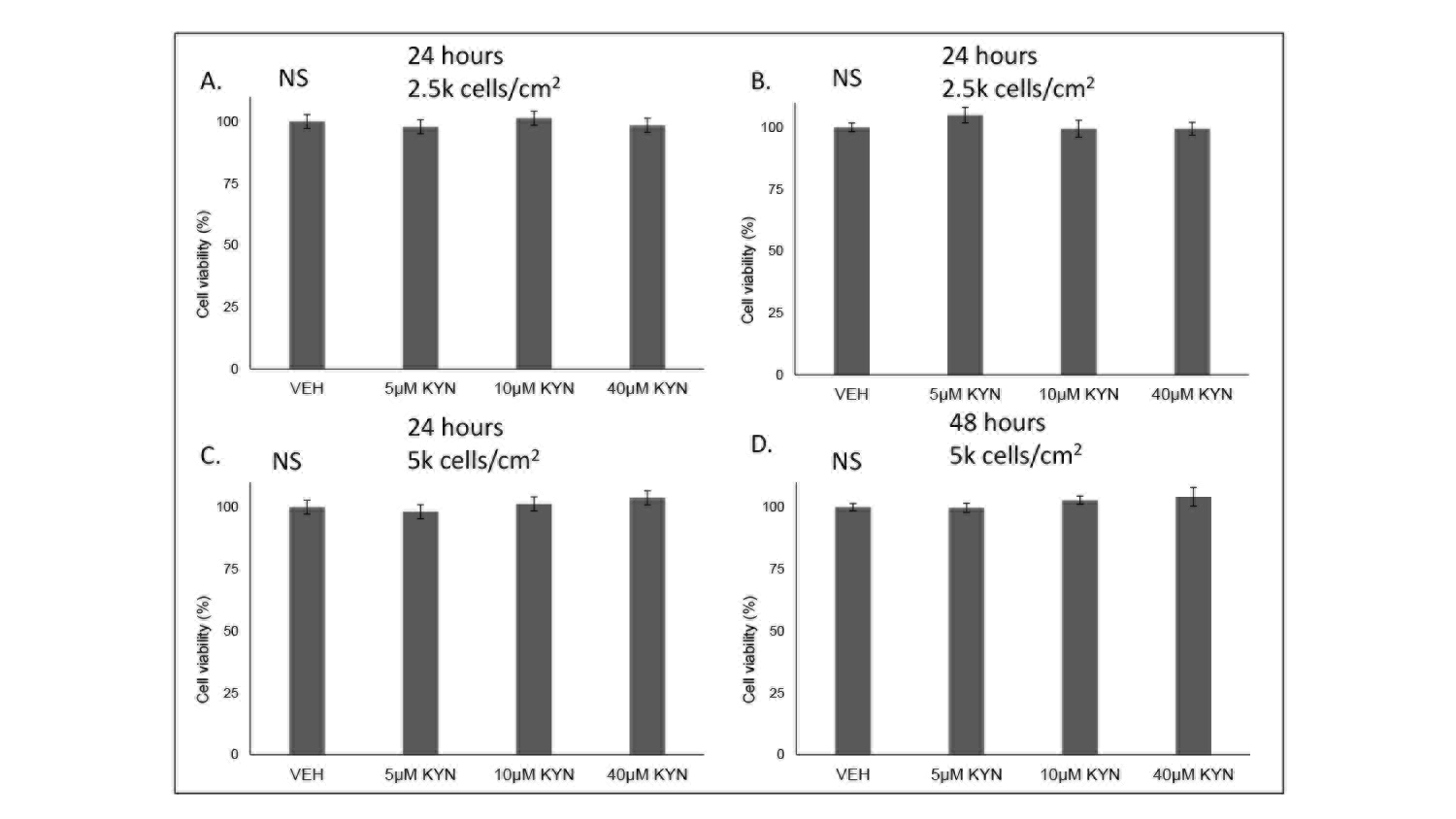

Supplement: Supplementary Materials — Supplemental Figure 1: the effect of KYN on C2C12 myoblast viability. (A–D) There was no change in C2C12 myoblast viability with a seeding density of 2500 cells/cm2 or 5000 cells/cm2 and 24 or 48 hours of treatment. [file 9894238.f1.tif]
